# Crystalline CO_2_-based polycarbonates prepared from *racemic* catalyst through intramolecularly interlocked assembly

**DOI:** 10.1038/ncomms9594

**Published:** 2015-10-15

**Authors:** Ye Liu, Wei-Min Ren, Wei-Ping Zhang, Rong-Rong Zhao, Xiao-Bing Lu

**Affiliations:** 1 State Key Laboratory of Fine Chemicals, Dalian University of Technology, Dalian 116024, China

## Abstract

The crystalline stereocomplexed polycarbonates can be prepared by mixing enantiopure polymers with opposite configuration, which derived from the asymmetric copolymerization with CO_2_ using enantiopure catalyst or/and chiral epoxides. Herein, we develop a powerful strategy for producing crystalline intramolecular stereocomplexed polycarbonates from *racemic* catalysts, which possess similar thermal stability and crystalline behaviour in comparison with the stereocomplexes by mixing opposite enantiopure polymers. Living polymer chains shuttle between catalyst molecules with different configurations to produce diastereomeric active species which is suggested to be responsible for the formation of isotactic multiblock polycarbonates in *racemic* bimetallic cobalt catalyst-mediated stereoselective copolymerization of CO_2_ and *meso*-epoxides. Solid-state NMR spectroscopy study suggests that the interaction in the carbonyl and methine regions is responsible for the strong crystallization capacity and compact package structure in the crystalline polycarbonates.

## Abstract (web summary)

Crystalline polycarbonates can be formed by mixing enantiopure polymers with opposite configurations. Here, the authors produce crystalline intramolecular stereocomplexed polycarbonates using a racemic catalyst, and show that these display similar properties to those formed by mixing the two enantiomers.

## Graphical abstract

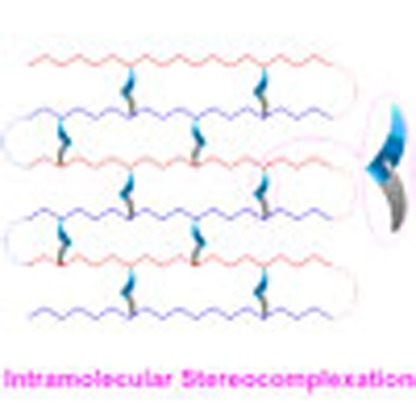

The alternating copolymerization of carbon dioxide (CO_2_) with epoxides to provide degradable polycarbonates is widely regarded as a promising green process worthy of intense scrutiny since it utilizes CO_2_ as C_1_ feedstock, an abundant and renewable carbon resource^1^,^2^,^3^,^4^,^5^,^6^. In the past decade, numerous homegeneous and hetereogeneous catalyst systems were developed for this transformation for achieving enhanced activity and high molecular weight^7^,^8^,^9^,^10^,^11^,^12^,^13^,^14^,^15^,^16^,^17^,^18^,^19^,^20^,^21^. Unfortunately, most of the previously reported CO_2_-based polycarbonates are amorphous, with a low glass transition temperature (*T*_g_) <50 °C, significantly confining their applications, especially as structural materials.

It is generally known that the physical properties of a polymer are determined not only by the monomer structure, its molecular weight and polydispersity, but also by the relative stereochemistry (the spatial arrangement of atoms or groups in a polymeric unit) of adjacent locations in the polymeric chains. A representative example is the widely studied polypropylene. The isotactic polypropylene is a typical semicrystalline material, possessing a melting temperature (*T*_m_) of 130−175 °C, dependent on the isotacticity, while the amorphous polypropylene is a viscous polymer at ambient temperature with a *T*_g_ of ∼0 °C (ref. 22). For CO_2_-based polycarbonates, in comparison with their amorphous structure, the crystalline forms should show improved thermal and mechanical properties, due to the high stereoregularity. In the recent contributions, some crystalline polycarbonates were prepared by stereospecific copolymerization of CO_2_ and epoxides using enantiopure metal-complex catalysts^23^,^24^,^25^. Notably, isotactic polycarbonates from *meso*-epoxides showed high levels of crystallinity, possessing *T*_m_s of 179−273 °C, dependent on the structure of the epoxides^26^,^27^,^28^,^29^,^30^. Interestingly, the cocrystallinzation of amorphous isotactic polycarbonates having opposite configurations and identical structures was observed to form crystalline stereocomplexes^31^,^32^, which show enhanced thermal stability and new crystalline behaviour, significantly distinct from the component enantiomers. These discoveries open up a new way to prepare various semicrystalline materials having a wide variety of physical properties. Nevertheless, these crystalline materials all originate from chiral isotactic polycarbonates prepared by the enantiopure metal-complex-mediated CO_2_/epoxides copolymerization. As a consequence, these processes are far away from practical applications, due to the high cost of chiral catalysts. Therefore, the exploration of the synthesis of crystalline CO_2_-based polycarbonates from *racemic* catalyst and *rac*- or *meso*-epoxides is highly desirable.

Herein, we report an approach for the synthesis of intramolecular stereocomplexed polycarbonates by stereoselective copolymerization CO_2_ with *meso*-epoxides using *racemic* dinuclear Co(III) complex as catalyst (Fig. 1), which possess similar thermal stability and crystalline behaviour in comparison with the stereocomplexes by mixing opposite enantiopure polymers.

## Results

### Synthesis of crystalline polycarbonates from 3,5-dioxaepoxide

**Figure 2:**
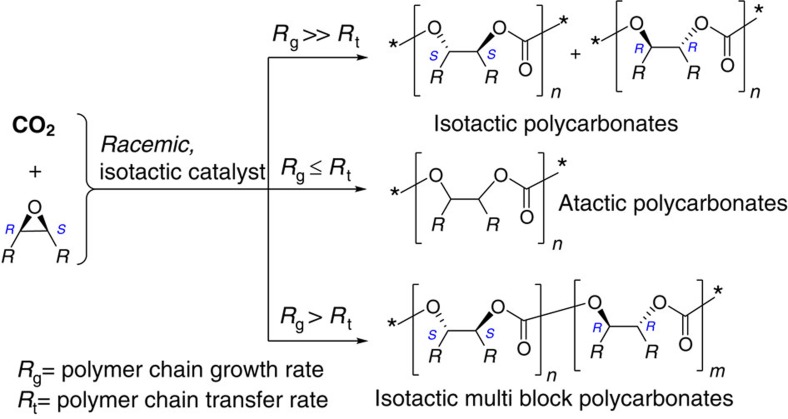
Effects of polymer chain growth and transfer rate on the polymer structure. Three possible microstructures produced from the alternating copolymerization of CO_2_ with *meso*-epoxides using *racemic* isotactic catalyst.

For the alternating copolymerization of CO_2_ with *meso*-epoxides mediated by *racemic* isotactic catalyst systems, three possible microstructures might be observed in the resultant copolymers, dependent on the copolymerization chain growth rate (*R*_g_) and polymer chain-transfer rate (*R*_t_) (Fig. 2). Only when *R*_g_ is significantly higher than *R*_t_, the *racemic* isotactic catalyst-mediated copolymerization reaction provides isotactic polycarbonates or isotactic multiblock polymers. The *racemic*–(SalBinap)–AlO^*i*^Pr complex has been demonstrated to be very effective for the ring-opening polymerization of *racemic* lactide, affording the crystalline, stereoblock polymers^33^. Coates and co-workers presented the first report for synthesizing a broad range of highly isotactic polyethers via the enantioselective polymerization of *racemic* epoxides using *racemic* catalyst^34^,^35^,^36^. In this system, (*S*)-binaphthol linked dinuclear cobalt complex predominantly catalysed the ring-opening polymerization of (*S*)-epoxides to afford (*S*)-polyethers, while the polymerization of (*R*)-epoxides only concerned (*R*)-binaphthol linked catalyst to provide (*R*)-polyethers. The resultant mixture of (*S*)- and (*R*)-polyethers are highly isotactic, and most of them display high *T*_m_ values. Nevertheless, no cocrystallization occurs in the resultant mixture, in comparison with (*S*)- or (*R*)-polyethers.

**Figure 3:**
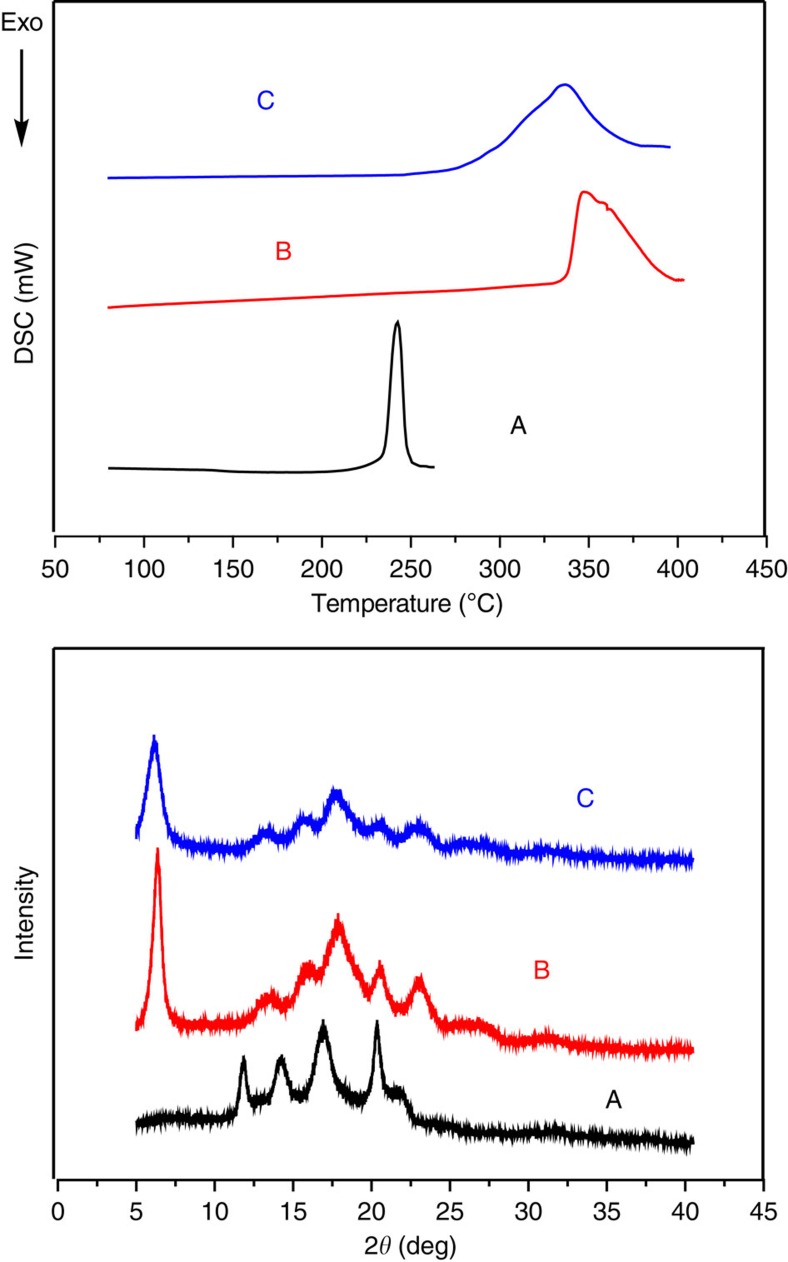
DSC thermograms and wide-angle X-ray diffraction profiles of various PCXCs. (A) (*R*)- or (*S*)-PCXC with 99% *ee*; (B) stereocomplexed PCXC prepared by mixing *(R*)- and (*S*)-polymres with 1:1 mass ratio; (C) PCXC prepared from *rac*-**1**/PPN–DNP catalysed CXO/CO_2_ copolymerization (Table 1, entry 1). The samples were crystallized isothermally at 180 °C for 2 h and samples of B and C in DSC thermograms was determined by FSC.

**Figure 4:**
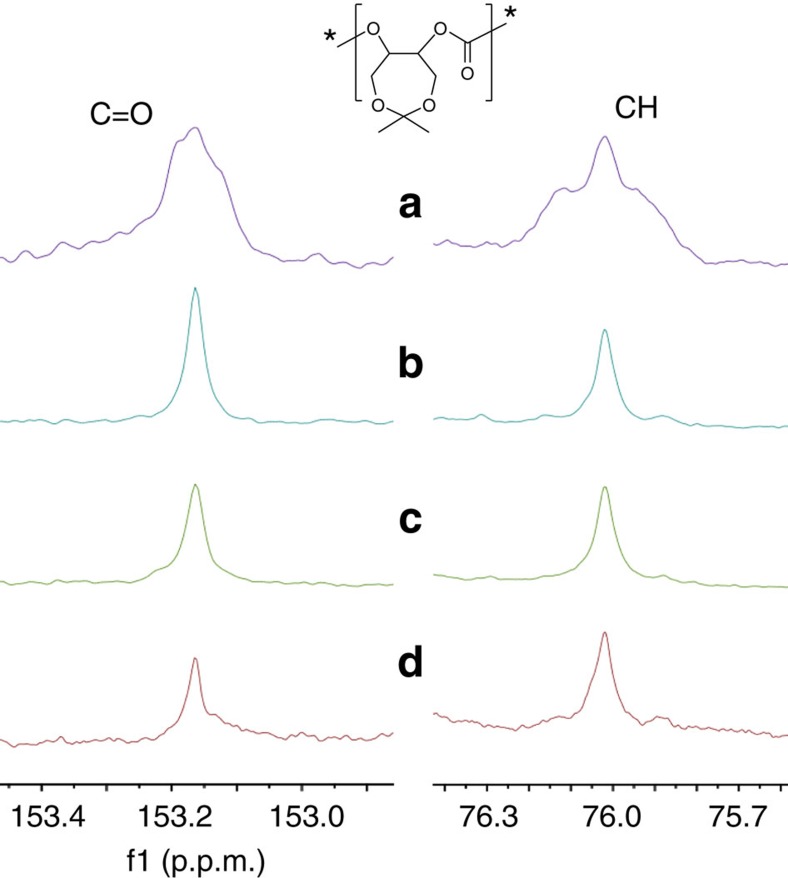
**The carbonyl and methine region of** ^**13**^**C NMR spectra of various PCXCs.** (**a**) Atactic PCXC; (**b**) enantiopure isotactic (*S*)-PCXC; (**c**) stereocomplexed PCXC prepared by mixing *(R*)- and (*S*)-polymers in 1:1 mass ratio; (**d**) PCXC prepared from *rac*-**1**/PPN–DNP mediated CXO/CO_2_ copolymerization (Table 1, entry 1).

Initially*, racemic* dinuclear Co(III) catalyst **1** was first applied to the alternating copolymerization of CO_2_ with 4,4-dimethyl-3,5,8-trioxabicyclo[5.1.0]octane (CXO), a *meso*-epoxide with high reactivity. Previous study demonstrated that isotactic polycarbonates from CXO/CO_2_ enantioselective copolymerization (PCXC) exhibited a melting temperature of 242 °C (ref. 28). Notably, when (*R*)-PCXC and (*S*)-PCXC are mixed in equivalent amounts, cocrystallization occurs, affording a stereocomplex with a new crystalline behaviour, significantly different from that of the sole configuration PCXC^32^. As a consequence, it was expected to form crystalline stereocomplex from *racemic* dinuclear Co(III) catalyst **1**-mediated CXO/CO_2_ copolymerization, if (*R,R,R,R*)-**1** in the *racemic* catalyst system predominantly produces enantiopure (*R*)-PCXC, and (*S,S,S,S*)-**1** mainly affords enantiopure (*S*)-PCXC. We delightedly found that the CO_2_/CXO copolymerization using *racemic*-**1** in conjunction with PPN–DNP (PPN, bis(triphenylphosphine)iminium; DNP, 2,4-dinitrophenoxide) at 25 °C and 1.5 MPa CO_2_ pressure yielded highly crystalline polymer with a turnover frequency of 199 h^−1^ (Table 1, entry 1). On the basis of fast-scan chip-calorimeter measurement (Flash DSC), high-melting endothermic peak was found at 340 °C, which is significantly different from the sole configuration (*R*)- or (*S*)-PCXC (Fig. 3, top, plots A and C). Also, the wide-angle X-ray diffraction study confirms its semicrystalline structure. Several diffraction peaks appearing at 2*θ* equal to 6.4°, 13.3°, 16.0°, 17.9°, 20.5° and 23.0° (*d*=13.80, 6.65, 5.53, 4.95, 4.33 and 3.86, respectively) are consistent with the stereocomplexed PCXC by mixing enantiopure isotactic (*R*)- and (*S*)-PCXC in equivalent amount, but significantly distinct from that of the individual enantiomers (Fig. 3, bottom). Moreover, ^13^C NMR study demonstrates that no obvious difference was observed in the peaks corresponding to carbonyl and methine region between PCXCs prepared by mixing opposite enantiomers and by using *rac*-**1**, but significantly different from the atactic analogue (Fig. 4).

Previously, we have demonstrated that isotactic (*R*)-PCXC or (*S*)-PCXC were easily dissolved in various organic solvent, such as dimethylsulphoxide and tetrahydrofuran (THF), while stereocomplexed PCXC prepared from mixing equivalent (*R*)- and (*S*)-PCXC had no solubility in these solvents. It was found that the copolymer formed from the *racemic* catalyst system also had no solubility in both dimethylsulphoxide and THF, suggesting the formation of the stereocomplex. Furthermore, methanol was added as a chain-transfer reagent to the copolymerization mediated by *rac*-**1**/PPN–DNP catalyst system at the identical reaction conditions. We discovered that the resultant polymers were also crystallizable, although the melting temperature was decreased to a certain extent (Table 1, entries 1–4). For example, a *T*_m_ of 221 °C was found in the copolymer produced from *rac*-**1**/PPN–DNP catalyst system in the presence of 100 equivalents of MeOH, which is 119 °C lower than the PCXC resulted from the same catalyst in the absence of MeOH (Supplementary Fig. 1). The addition of methanol also resulted in the significant decrease in the copolymerization rate. In addition, a decrease in *M*_n_ is very obvious, and thereby causing their dissolvable in THF and dimethylsulphoxide. ^13^C NMR analysis show that the peaks corresponding to carbonyl and methine region were found to be splitted when the reaction was carried out in the presence of methanol (Supplementary Fig. 2), suggesting a decrease in stereoregularity.

It is worth noting that the intensities of various diffraction peaks in the copolymer sample from *racemic* catalyst are obviously lower than the stereocomplexed PCXC obtained from the 1:1 mixture of the opposite enantiomers (Fig. 3, bottom, plots B and C). In addition, the melting endothermic peak is also slightly lower than that of the stereocomplexed PCXC with a *T*_m_ of 347 °C (Fig. 3, top, plots B and C). These results suggest that the crystallinity of the resultant PCXC from *racemic*-**1** catalyst system is significantly lower than that of the stereocomplexed PCXC consisted of the mixed opposite enantiomers. We tentatively assume that the copolymer from *racemic*-**1** catalyst system is an isotactic multiblock polymer. The reduced isotacticity originates from the stereoerrors in the copolymer caused by the polymer growth-chain transfer between (*R,R,R,R*)-**1** and (*S,S,S,S*)-**1** during the copolymerization. As a result, the intramolecular cocrystallization of the isotactic multiblock-PCXC from *racemic*-**1** catalyst system predominantly contributes the formation of intramolecular stereocomplexed polycarbonates.

**Figure 5:**
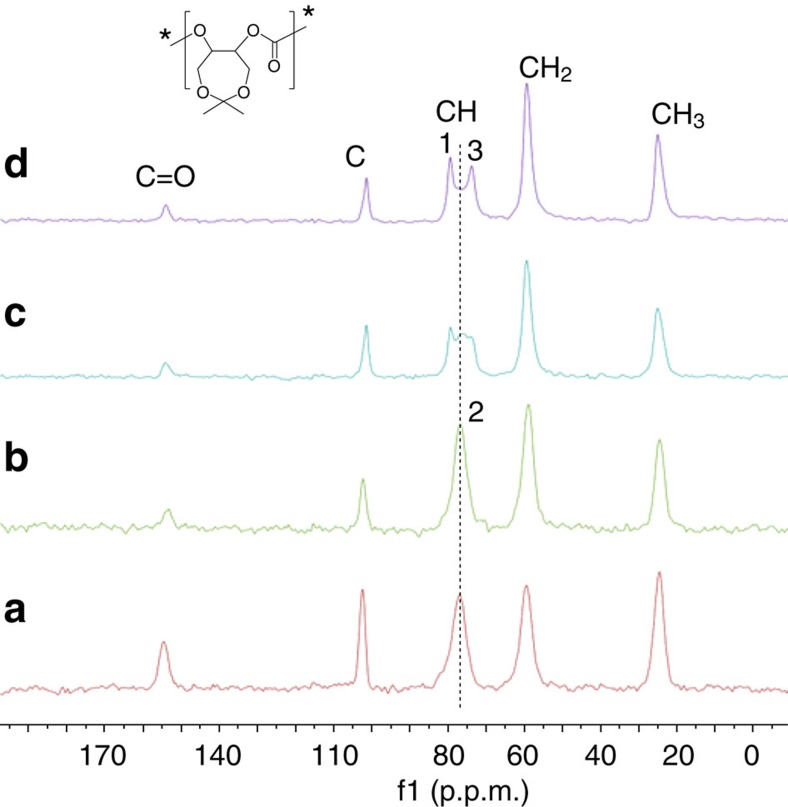
^**13**^**C CPMAS NMR spectra of various PCXCs.** (**a**) amorphous-PCXC; (**b**) enantiopure (*S*)-PCXC; (**c**) isotactic multiblock-PCXC; (**d**). stereocomplexed-PCXC.

Solid state NMR spectroscopy is a powerful tool for studying the polymer segment, structure and dynamics. Macromolecular motions covering a wide range of time scales have long been considered to affect the physical and mechanical properties^37^,^38^,^39^,^40^. Usually, polymers in amorphous state show high-amplitude motions, especially for the temperature above the *T*_g_s. As previously mentioned, there are huge differences in physical properties for the amorphous, enantiopure and stereocomplexed-PCXCs in solid state, such as solubility, melting and crystalline behaviour. In the present study, solid state NMR spectroscopy was also employed for studying the difference in microstructure of various PCXCs (Fig. 5). Spin-lattice relaxation time (*T*_1_) was measured for each carbon atom in four representative PCXC samples under the cross-polarisation condition by application of the saturation recovery-based sequence (Table 2). We discovered that the *T*_1_ value of carbonyl region for the enantiopure PCXC was longer than the amorphous state. Especially, the *T*_1_ value of carbonyl region for the stereocomplexed PCXC is up to 223 s, which is 175 s longer than the amorphous state and 131 s longer than enantiopure-PCXCs, in accordance with its much stronger crystallization capacity and compact package structure. Notably, it was demonstrated that PCXC resulted from *racemic*-**1** (multiblock-PCXC) possessed a similar structure with the stereocomplexed PCXC, because its *T*_1_ value in carbonyl region also reached to 156 s. There is an interesting information for methine region, which was found to be splitted to three peaks for multiblock-PCXCs, and two peaks for stereocomplexed-PCXCs. The *T*_1_ values are 165 and 150 s for stereocomplexed-PCXCs, in agreement with 110 and 116 s for multiblock-PCXCs. However, no split was observed in the methine region for amorphous or enantiopure-PCXCs, in which *T*_1_ values are 38 and 36 s, respectively. Interestingly, *T*_1_ value for the middle peak in the methine region for multiblock-PCXCs is 50 s, corresponding to the methine region for amorphous or enantiopure-PCXCs. We tentatively ascribe it to the minor glassy state in the crystalline domains, which originated from the stereoerrors in the copolymer caused by the polymer growth-chain transfer between (*R,R,R,R*)-**1** and (*S,S,S,S*)-**1** during the copolymerization. Nevertheless, *T*_1_ values of quaternary and primary carbon for amorphous PCXCs were very similar to those measured for stereocomplexed- and multiblock-PCXCs, indicating that they are more inclined to motion and the energy can be released more easily.

### Mechanistic study for *racemic*-1 mediated copolymerization

Indeed, ^13^C NMR spectra of carbonyl and methine regions in Fig. 4 did not give the accurate isotacticity of the multiblock-PCXCs originated from the *racemic* catalyst system. In order to confirm the formation of the isotactic multiblock structure, cyclohexene oxide (CHO) was chosen as a model monomer of *meso*-epoxides for testing the stereoregularity of its CO_2_ copolymer produced by *racemic* Co(III) complexes, since the microstructure of poly(cyclohexene carbonate) (PCHC) was well-characterized^41^,^42^,^43^. Indeed, CHO also has relatively high reactivity in copolymerizing with CO_2_ catalysed by both mono- and di-nuclear Co(III) complexes in the presence of a nucleophilic cocatalyst. In previous study, we have demonstrated that for the mono-nuclear Co(III) complex-mediated CO_2_/epoxide copolymerization, the dissociation of the propagating carboxylate from the metal centre is a much faster process than propagation, and the free propagating carboxylate can also act as a nucleophile for attack at a cobalt-coordinated epoxide, so the binary catalyst system of *racemic* mono-nuclear Co(III)–Salen complex and PPN–DNP for CHO/CO_2_ copolymerization provided atactic PCHC^18^. On the contrary, the *racemic* dinuclear Co(III) complex **1** gave isotactic-enriched PCHC, based on the ^13^C NMR analysis. However, the isotacticity is obviously lower than that obtained from enantiopure dinuclear Co(III) complex. This means the occurrence of the copolymer-chain transfer between two kinds of catalyst molecules with different configurations.

**Figure 6:**
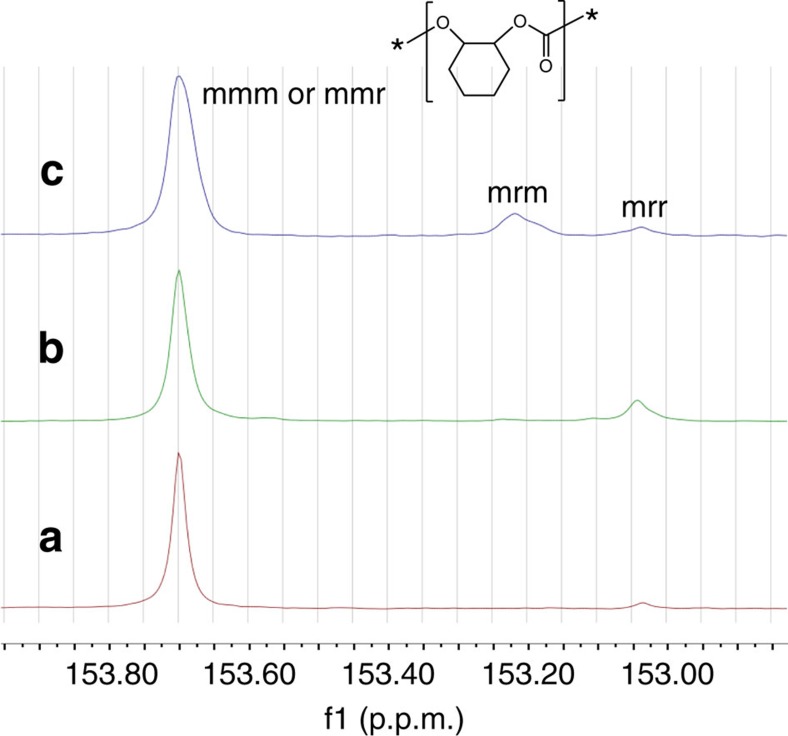
**Carbonyl region of the** ^**13**^**C NMR spectra (125 MHz, CDCl**_**3**_**) of various PCHCs.** (**a**) PCHC with 0.96 *P*_m_ catalysed by enantiopure dinuclear Co(III) catalyst in toluene^27^; (**b**) PCHC with 0.84 *P*_m_ catalysed by enantiopure dinuclear Co(III) catalyst^27^; (**c**) PCHC with 0.82 *P*_m_ catalysed by catalyst *rac*-**1**/PPN–DNP (Table 1, entry 7).

Previously, various stereoregular PCHCs tetrad and triad sequences have been assigned in the ^13^C NMR spectrum^42^,^43^. In keeping with the previously established conventions in this field, it is important to note that [m] and [r] assignments used herein represent the relative stereochemistry of the carbons of the cyclohexene carbonate units (Supplementary Fig. 3). By synthesizing model poly(cyclohexene carbonate) oligomers or using Bernoullian statistical methods, all [mmm] and [mmr] tetrads were correlated to one central resonance at 153.7 p.p.m. and the remaining *r*-centred tetrads resided in the 153.3–153.0 p.p.m. range. The carbonyl region of the ^13^C NMR spectra of various PCHCs resulted from different catalysts or conditions is shown in Fig. 6 (The relationship between the tetrad sequences and the polymer microstructures was described in Supplementary Fig. 3). On the basis of the peaks assigned to the appropriate tetrads in accordance with the literature, the PCHC with a *P*_m_ of 0.84 obtained from (*S,S,S,S*)-**1**/PPN–DNP catalyst system revealed a ^13^C NMR spectrum with two distinct resonances at 153.70 and 153.04 p.p.m. assigning to [mmm+mmr] and [mrr] tetrads, respectively (Fig. 6, plot B). Especially, the peak at 153.04 p.p.m. for PCHC with a *P*_m_ of 0.96 decreased significantly (Fig. 6, plot A). The *r*-centred [mrr] tetrad was produced by the errors in the chain growth (mismatched monomer was incorporated) and then corrected by the chiral environment that is constructed by the ligand around the metal centre through an enantiomorphic site control. However, for polycarbonates resulted from *racemic*-**1** (Fig. 6, plot C), a peak at 153.22 p.p.m. was discovered, corresponding to [mrm] tetrad, significantly distinct from PCHC resulted from (*S,S,S,S*)-**1** with the same *P*_m_s (Fig. 6, plot B). Moreover, because of the formation of [mmr] tetrads, the peak corresponding to m-centred tetrads of polycarbonates resulted from *racemic*-**1** become broaden in comparison with the PCHC with the identical stereoregularity. The ^13^C NMR spectra of methylene region also confirmed the results (Supplementary Fig. 4). The presence of a small [mrr] peak also suggests that a minimal amount of the unpreferred enantiomer is incorporated into the chain at a level, consistent with the PCHCs described in plots A and B.

On the basis of [mrm] tetrad in ^13^C NMR analysis, we can conclude that the polymer should have -*RRRRRRSSSSSS*- or -*SSSSSSRRRRRR*- sequences in the main chain. In fact, the polycarbonates produced from *racemic*-**1** has a stereo multiblock structure with alternating blocks of (*R*)- and (*S*)-polymer segments, rather than a stereocomplex of two highly enantiomerically enriched chains. A statistical model was used to simulate the spectrum of the PCHC with stereochemical defects formed in the polymer growth-chain transfer between (*R,R,R,R*)-**1** and (*S,S,S,S*)-**1**, suggesting that a block in the stereo multiblock PCHC contain an average of five enantiomerically pure carbonate units.

In the recent contributions, we demonstrated that the enantiopure biphenol-linked dinuclear Co(III) complex **1** was a privileged chiral catalysts for asymmetric copolymerization of CO_2_ with various *meso*-epoxides, showing high activity and excellent enantioselectivity^27^,^28^,^29^. The mechanistic study revealed that chain-growth step predominantly involves an intramolecular bimetallic cooperation mechanism, wherein alternating chain growth and dissociation of propagating carboxylate species takes turn between two Co(III) ions from the inside cleft of dinuclear Co(III) catalysts by the nucleophilic attack of the growing carboxylate species at one metal centre towards the activated epoxide at the other^44^. It was also found that the propagating polymer chain transfer could be caused by protic solvents such as water and methanol, rather than the excess cocatalyst. Although every effort has been made to keep the copolymerization reaction anhydrous, we were concerned that trace quantities of water might be present, and thereby cause the growing polymer-chain transfer. Furthermore, it was found that the addition of 10 equiv. of methanol resulted in the significant decrease in copolymer molecular weight from 32.0 to 10.1 kg mol^−1^, and a slight loss in *P*_m_ from 0.82 to 0.79 (Table 1, entry 8).

**Figure 7:**
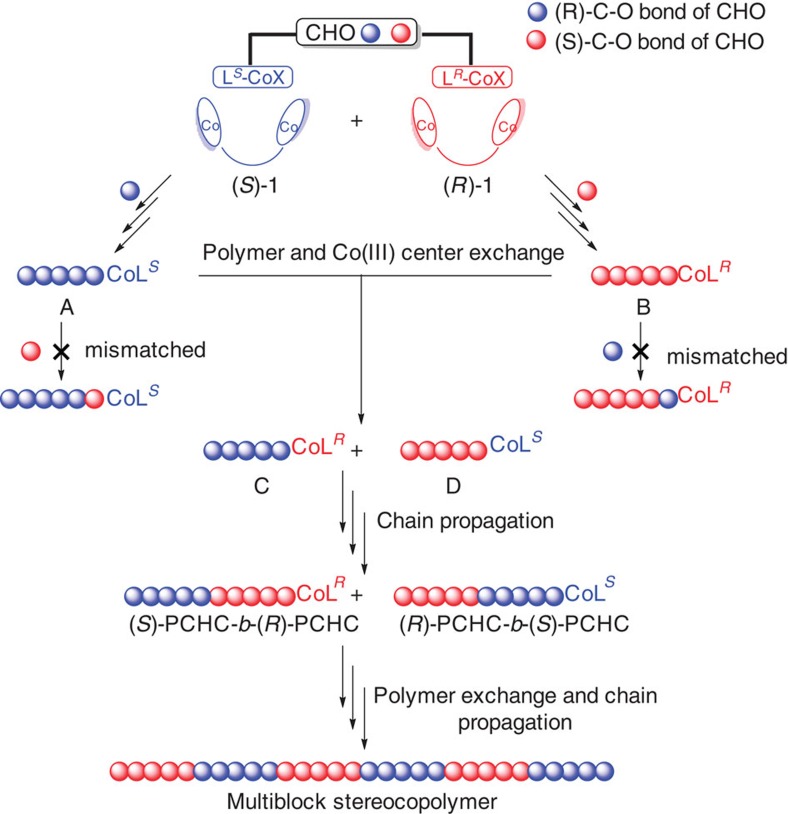
Possible mechanism for the formation of isotactic multiblock polycarbonates. *Racemic* dinuclear Co(III) mediated CHO/CO_2_ copolymerization was selected as a model reaction.

On the basis of the evidence and analysis mentioned above, the possible formation process for isotactic multiblock PCHC is proposed in the Fig. 7. Since the insertion of CO_2_ into the growing polymer chain is a fast process, the predominant ring-opening event is the reactions of (*S*)-**1** with (*R*)-C−O and (*R*)-**1** with (*S*)-C−O bond of CHO, affording (*S*)-PCHC and (*R*)-PCHC, respectively (enantiomeric active species **A** and **B**). However, the adventours water or the addition of protic solvent probably results in polymer-chain exchange between the (*R*)-PCHC anchored on (*R*)-**1** and the (*S*)-PCHC anchored on (*S*)-**1**, affording the (*S*)-PCHC anchored on (*R*)-**1** and the (*R*)-PCHC anchored on (*S*)-**1** (diastereomeric active species **C** and **D**). At this point, polymer chain propagation resumes with the favoured stereoisomer, creating a diblock structure. The polymer chain exchange and propagation take place repeatedly to provide the isotactic multiblock polycarbonates.

It is a pity that the isotactic multiblock PCHC with a *P*_m_ of 0.82 was amorphous material with a *T*_g_ of 125 °C, slightly higher than the atactic PCHC. Indeed, no crystallizability of the isotacticity-enriched PCHC is not strange. In previous contribution, we have demonstrated that only PCHCs with more than 90% isotacticity were crystallizable^26^. However, we expect with great passion the formation of the intramolecular stereocomplexed PCHC, since a stereocomplex formed by the polymer assembly of optically active PCHCs with opposite configurations was previously confirmed^30^. In order to validate our supposition, solid state NMR spectroscopy was also employed for studying the difference of the microstructure of various PCHCs. (Supplementary Fig. 5 and Supplementary Table 1). As anticipated, the *T*_1_ value of carbonyl region for the stereocomplexed PCHC is up to 271 s, which is 223 s longer than that for the amorphous PCHC. Similarly, the *T*_1_ values of methine (250 and 195 s) and methylene carbons (146 and 110 s) for the stereocomplexed PCHC are significantly longer than that for the amorphous PCHC (35 s for methine region, and 23 and 21 s for methylene carbons). However, for the isotactic multiblock PCHC, the *T*_1_ values of carbonyl region and methine carbons are 56 and 39 s, respectively, while that of methylene carbons are 26 and 24 s. These values are slightly higher than that for the amorphous state, but significantly lower than that for the stereocomplexed PCHC.

## Discussion

In conclusion, novel intramolecular stereocomplexed polycarbonates were synthesized by the stereoselective copolymerization of CO_2_ and *meso*-epoxides using *racemic* bimetallic cobalt catalyst system. Highly enantioselective chain growth in an enantiopure catalyst molecule and the copolymer-chain transfer between different configuration catalyst molecules results in the formation of the isotactic multiblock polycarbonates. Solid state NMR spectroscopy study suggests that the interaction in the carbonyl region is responsible for the strong crystallization capacity and compact package structure in the crystalline polycarbonates. This is the only example for the synthesis of crystalline CO_2_ polymers from *racemic* catalyst. Due to the use of the inexpensive *racemic* or achiral ligand, the present synthesis strategy is of great importance for preparing various intramolecular stereocomplexed polycarbonates with enhanced thermal stability.

## Methods

### General

All manipulations involving air- and/or water-sensitive compounds were carried out in a glove box or with the standard Schlenk techniques under dry nitrogen. CO_2_ (99.995%) was purchased from Dalian Institute of Special Gases and used as received. Methylene chloride and chloroform were distilled from calcium hydride under nitrogen. Tetrahydrofuran and toluene were distilled from sodium/benzophenone under nitrogen. Epoxides were purchased from Acros and distilled over calcium hydride.

### Fast-scan chip calorimeter

Fast-scan chip calorimetry (FSC) was performed with the commercialized FSC (Flash DSC1, Mettler-Toledo, Switzerland). The empty chip-sensor was calibrated according to the standard procedure before the experiment. The ready temperature of test module for all measurements was set as 30 °C. Purge nitrogen gas was used as the protection atmosphere with the constant flow rate 50 ml min^−1^. First cycle: from 20 °C to 280 °C at a heating rate of 3,000 K s^−1^, and holding at 280 °C for 5 min, and from 280 to 20 °C at a cooling rate of 3,000 K s^−1^. Second cycle: from 20 °C to 400 °C at a heating rate of 3,000 K s^−1^. For all FSC analysis, the result was given based on second cycle.

### Solid state NMR experiments

Solid state NMR experiments were performed using an Agilent DD2-500 MHz NMR spectrometer in 4-mm ZrO_2_ rotors at MAS frequencies ranging from 12 to 14 kHz. ^13^C cross-polarisation MAS NMR spectra were collected at 125 MHz with a B1(^13^C) field nutation frequency of 100 kHz, a contact time of 3 ms and a recycle delay of 4 s. ^13^C spin-lattice relaxation experiments were carried out under CPMAS conditions using the saturation recovery-based sequence. The chemical shifts were referenced to the adamantane with the upfield methine peak at 29.5 p.p.m.

Details of other experiments see Supplementary Methods.

## Additional information

**How to cite this article:** Liu, Y. *et al.* Crystalline CO_2_-based polycarbonates prepared from racemic catalyst through intramolecularly interlocked assembly. *Nat. Commun.* 6:8594 doi: 10.1038/ncomms9594 (2015).

## Supplementary Material

Supplementary Information Supplementary Figures 1-5, Supplementary Table 1, Supplementary Methods and Supplementary References

## Figures and Tables

**Figure 1:**
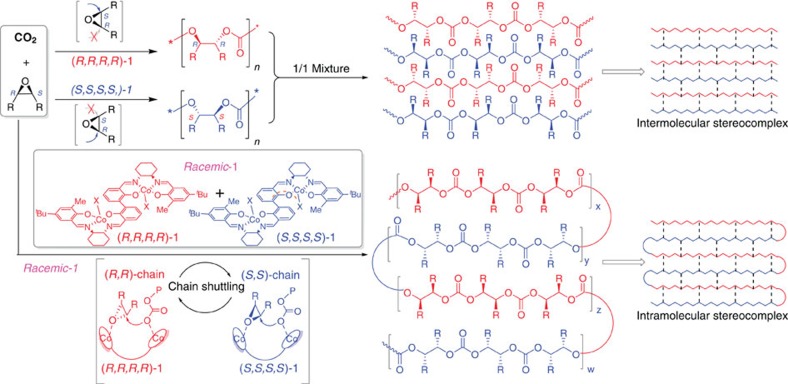
Synthesis of stereocomplexed polycarbonates. The difference in the synthetic routes of intramolecular and intermolecular stereocomplexes from copolymerization of CO_2_ with *meso*-epoxides.

**Table 1:**
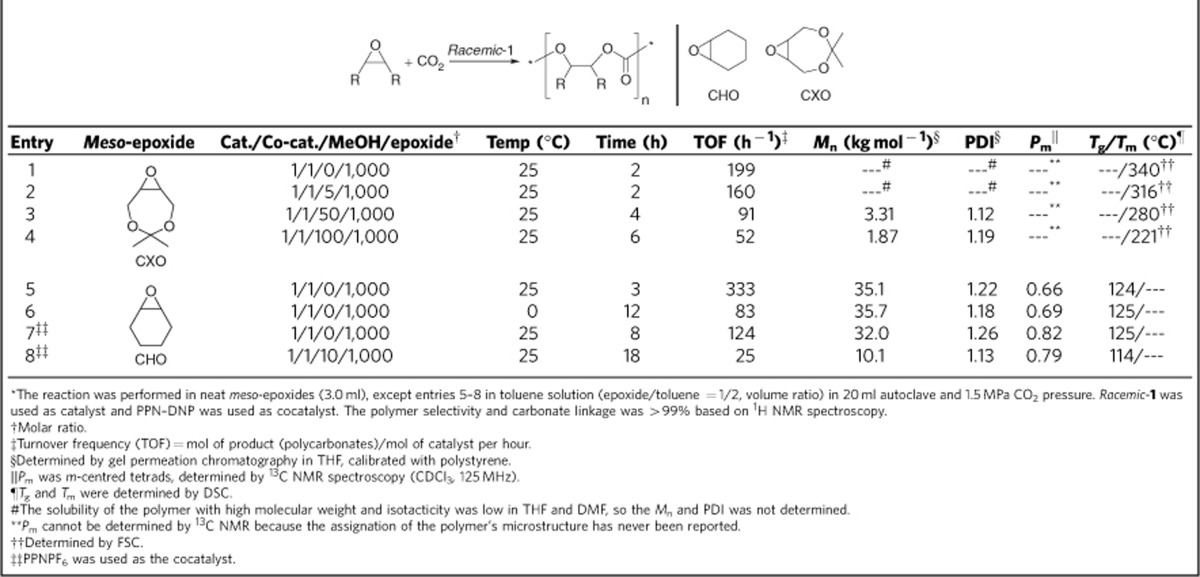
Enantiopure Co(III)-complex-mediated asymmetric CO_2_/*meso*-epoxides copolymerization*.

**Table 2:** ^13^C *T*_1_ relaxation times for solid various PCXCs at ambient temperature*.

**Entry**	**PCXC structure**	***T***_**1**_ **(s)**						
		**C=O**	**C**	**CH**			**CH**_**2**_	**CH**_**3**_
				**1**	**2**	**3**		
1	Amorphous	48	21	—	38	—	26	1.3
2	(*S*)-enantiopure	92	29	—	36	—	21	1.6
3	Multiblock	156	40	110	50	116	58	1.3
4	Stereocomplexed	223	42	165	—	150	81	1.6

^*^Measured with CPMAS NMR using the saturation recovery-based sequence.
