# Supplementary material for: Crystalline CO2-based polycarbonates prepared from racemic catalyst through intramolecularly interlocked assembly
Source: Nat Commun. 2015 Oct 15;6:8594. doi: 10.1038/ncomms9594 (PMC4634215; doi:10.1038/ncomms9594)
Supplement: Supplementary Information — Supplementary Figures 1-5, Supplementary Table 1, Supplementary Methods and Supplementary References [file ncomms9594-s1.pdf]

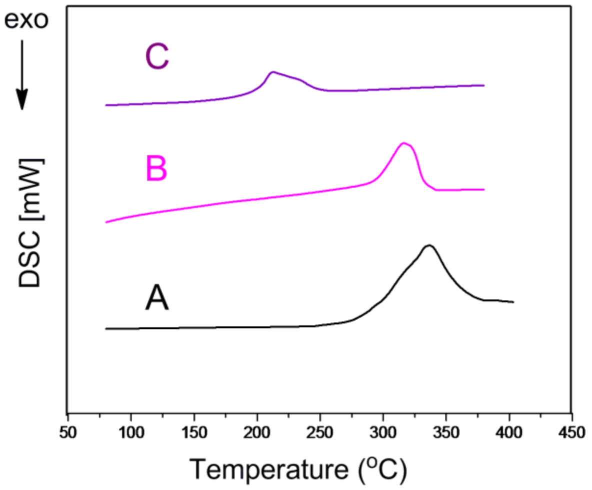

Supplementary Figure 1 | FSC thermograms of various PCXCs. A) PCXC prepared from *rac*-**1**/PPN-DNP catalyzed CXO/CO_2 copolymerization (Table 1, entry 1); B) PCXC prepared from *rac*-**1**/PPN-DNP catalyzed CXO/CO_2 copolymerization in presence of MeOH (*rac*-**1**/MeOH = 1/5, molar ratio) (Table 1, entry 2); C) PCXC prepared from *rac*-**1**/PPN-DNP catalyzed CXO/CO_2 copolymerization in presence of MeOH (*rac*-**1**/MeOH = 1/100, molar ratio) (Table 1, entry 4). The samples were crystallized isothermally at 180 ^{o}C for 2 h.

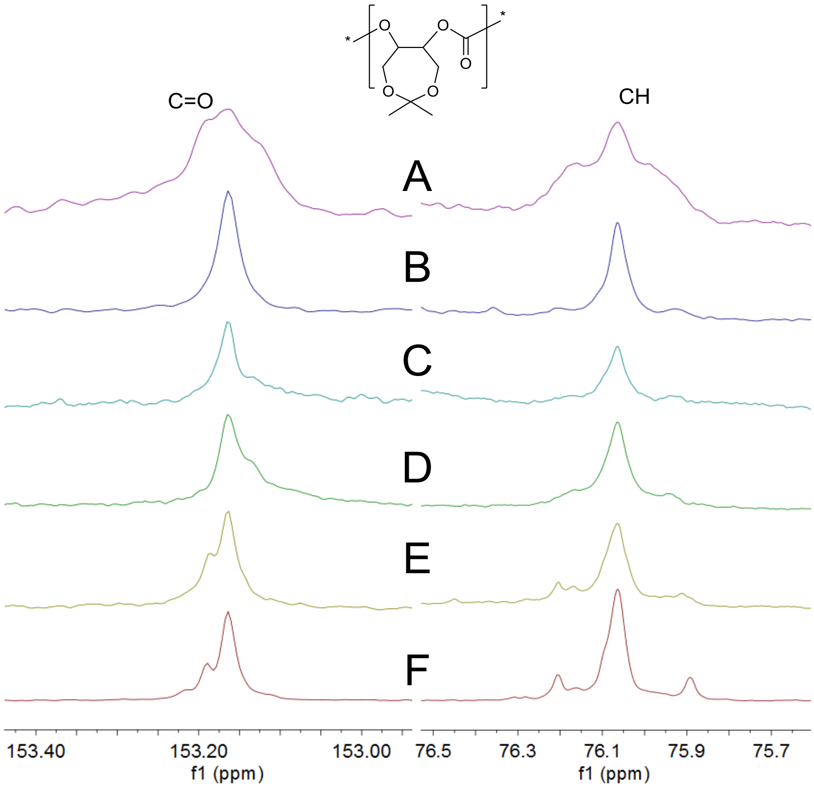

Supplementary Figure 2 | The carbonyl (left) and methine (right) region of ^{13}C NMR spectra of various polycarbonates. A) atactic PCXC; B) stereo-complexes result from (*R*)- and (*S*)-PCXCs with 1:1 mass ratio; (C) PCXC result from *rac*-**1**/PPN-DNP catalyzed CXO/CO_2 copolymerization; (D) PCXC result from *rac*-**1**/PPN-DNP catalyzed CXO/CO_2 copolymerization in presence of MeOH (*rac*-**1**/MeOH = 1/5, molar ratio); (E) PCXC result from *rac*-**1**/PPN-DNP catalyzed CXO/CO_2 copolymerization in presence of MeOH (*rac*-**1**/MeOH = 1/50, molar ratio); (F) PCXC result from *rac*-**1**/PPN-DNP catalyzed CXO/CO_2 copolymerization in presence of MeOH (*rac*-**1**/MeOH = 1/100, molar ratio).

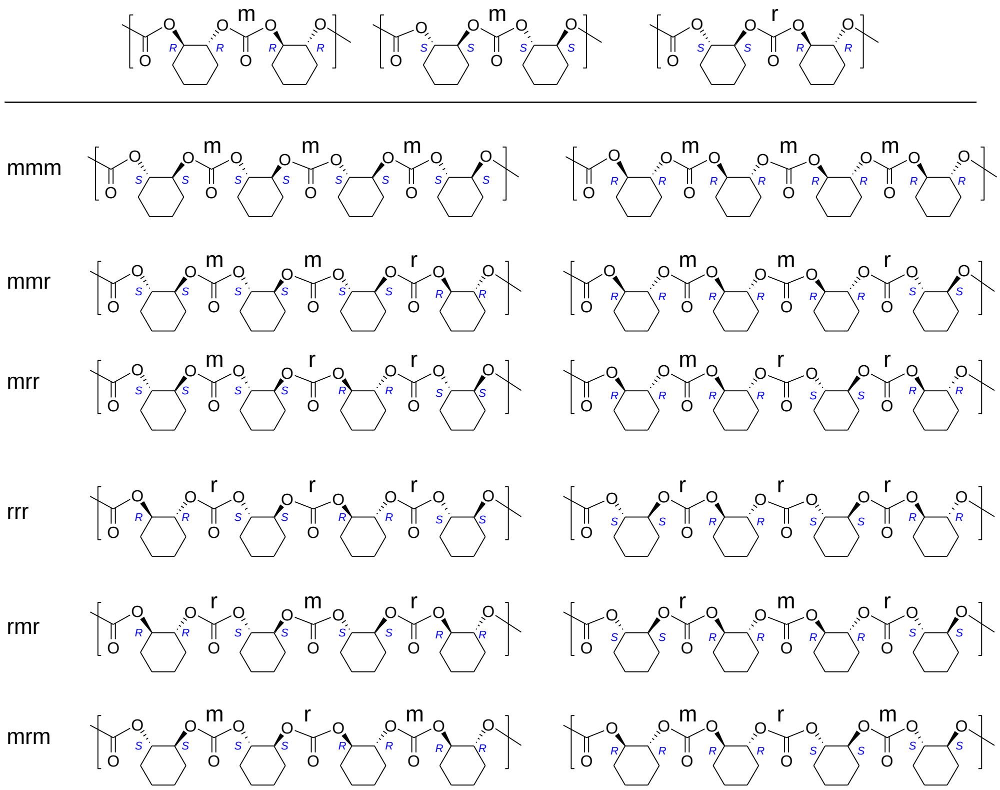

Supplementary Figure 3 | Various microstructures of poly(cyclohexene carbonate). The relationship between the tetrad sequences and the polymer microstructures.

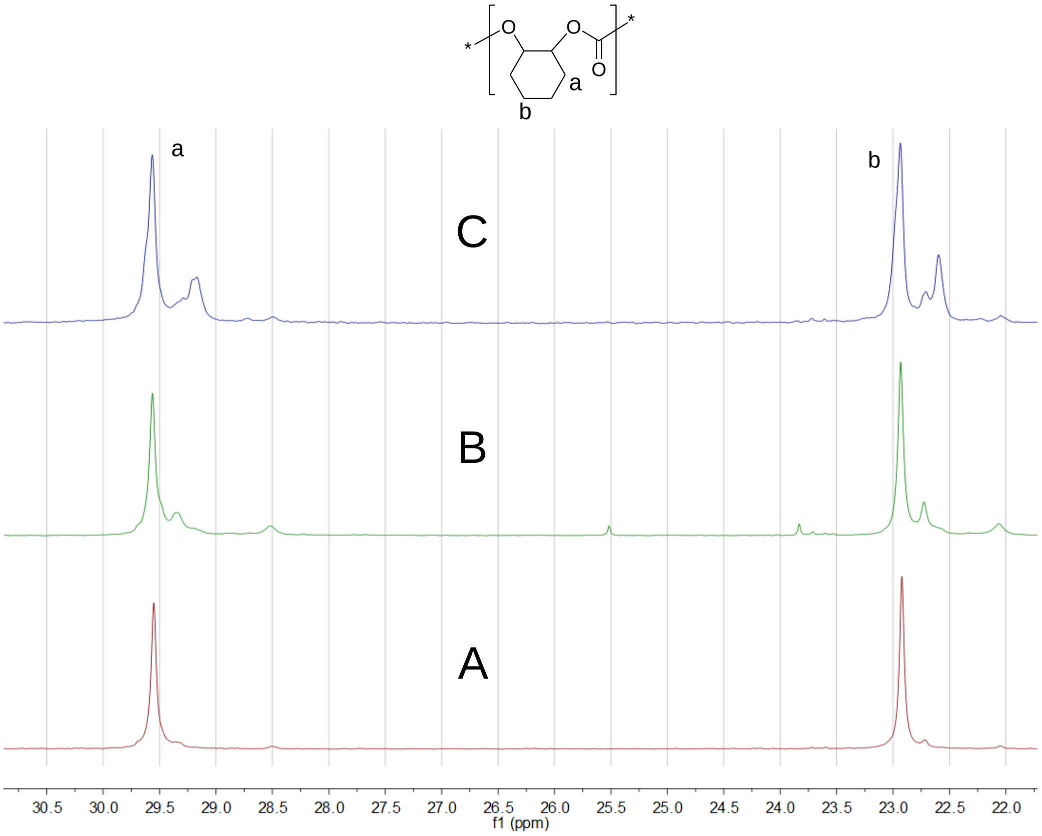

Supplementary Figure 4 | Methylene region of the ^{13}C NMR spectra (125 MHz, $CDCl_3$) of various PCHCs. A) PCHC with 0.96 P_m catalyzed by enantiopure dinuclear Co(III) catalyst in toluene;[1] B) PCHC with 0.84 P_m catalyzed by enantiopure dinuclear Co(III) catalyst;[1] C) PCHC with 0.82 P_m catalyzed by catalyst *racemic*-**1** (Table 1, entry 7).

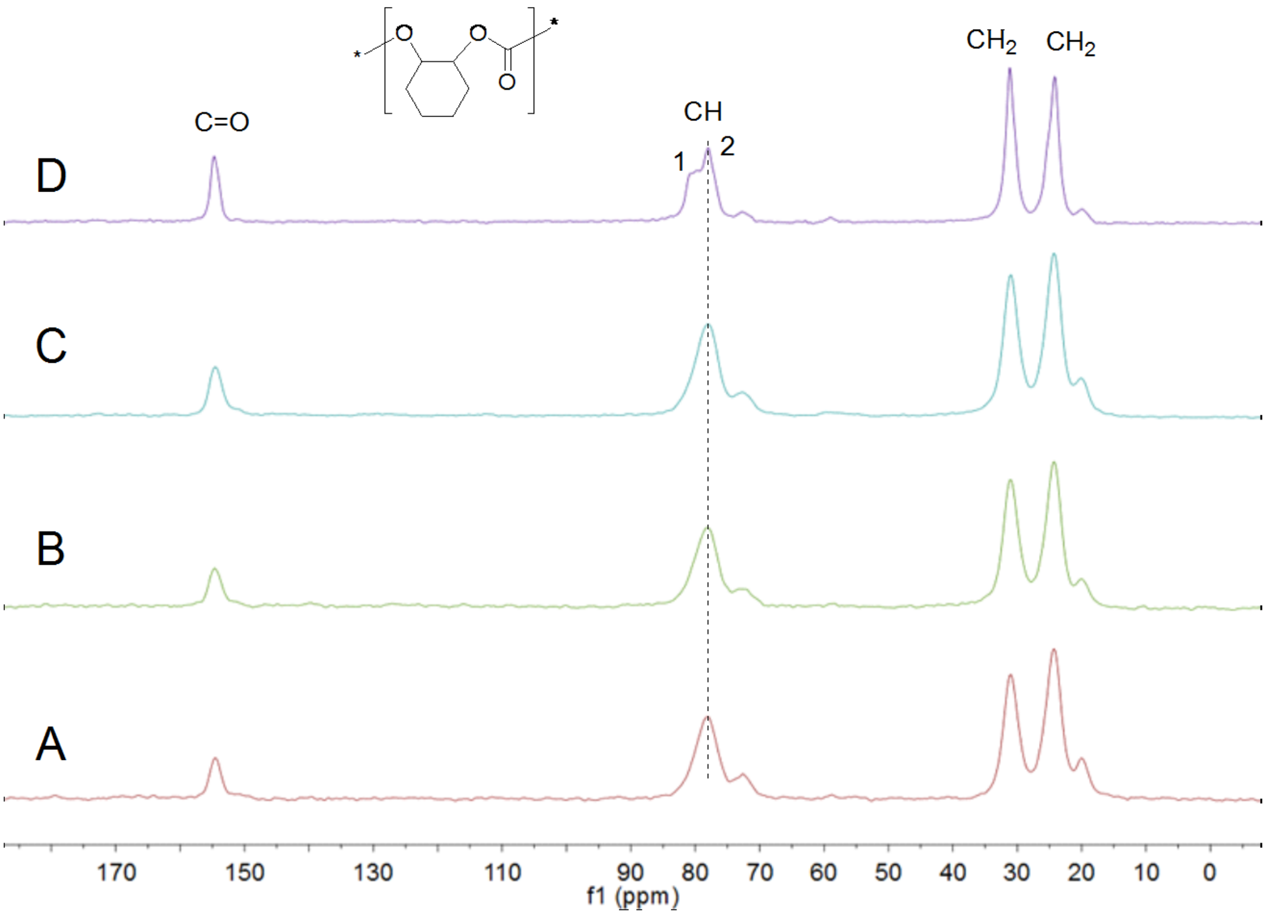

Supplementary Figure 5 | ^{13}C CPMAS NMR spectra of various PCHCs. A) amorphous-PCHC; B) (*S*)-enantiopure-PCHC with 0.84 P_m; C) Multiblock-PCHC; D) Stereocomplexed-PCHC.

Supplementary Table 1 | ^{13}C T_1 relaxation times for solid various PCHCs at ambient temperature[a]

Entry	Various PCXCs	T_1 (s)				
		C=O	CH		CH_2	CH_2
			1	2		
1	Amorphous-PCHC	48	---	35	23	21
2	PCHC with 0.84 P_m	44	---	32	22	20
3	Multiblock-PCHC	56	---	39	26	24
4	Stereocomplexed-PCHC	271	250	195	146	110

[a]Measured with CPMAS NMR using the saturation-recovery based sequence .

Supplementary Methods.

Procedures for the copolymerization of CO_2 with *meso*-epoxides: In a predried 20 mL autoclave equipped with a magnetic stirrer, catalyst *rac*-**1** (0.030 mmol, 1 equiv), cocatalyst (0.030 mmol, 1 equiv) and *meso*-epoxide (30 mmol, 1000 equiv) were dissolved in toluene in an argon atmosphere. After CO_2 was introduced, the reaction mixture was stirred at a desired temperature for an appropriate time. Then CO_2 was released, a small amount of the resultant mixture was removed from the autoclave for ^{1}H NMR analysis to quantitatively give the selectivity of polycarbonate to cyclic carbonate, as well as carbonate linkages. The crude polymer was dissolved in a 10 mL $CHCl_3$/MeOH (5/1, v/v) mixture with 0.5% HCl solution and precipitated from methanol. This process was repeated 3-5 times to completely remove the catalyst, and white polymer was obtained by vacuum drying.

NMR experiments. ^{1}H and ^{13}C NMR spectra were recorded on Varian INOVA-400 MHz type (^{1}H, 400 MHz), and a Bruker Avance 500 MHz type (^{13}C, 125 MHz) spectrometer, respectively. Their peak frequencies were referenced *versus* an internal standard (TMS) shifts at 0 ppm for ^{1}H NMR and against the solvent, chloroform-*d* at 77.0 ppm for ^{13}C NMR, respectively.

Mass spectrometry. A Micromass Q-Tof (Micromass, Wythenshawe, UK) mass spectrometer equipped with an orthogonal electrospray source (Z-spray) used for the cobalt complexes in positive ion mode (Capillary = 2000 V, Sample cone = 20 V).

Gel permeation chromatography (GPC). Molecular weights and molecular weight distributions of polymers were determined with a PL-GPC 220 high temperature chromatograph (Polymer Laboratories Ltd.) equipped with the HP 1100 series pump from Agilent Technologies. The GPC columns were eluted with tetrahydrofuran at 35 °C at 1.00 mL/min. The sample concentration was about 0.1%, and the injection volume was 100 μL. The curve was calibrated using monodisperse polystyrene standards covering the molecular weight rage from 580 to 460000 Da.

Differential scanning calorimetry (DSC) was carried out with a NETZSCH DSC 206 thermal analyzer. First cycle: from 20 °C to 180 °C at a heating rate of 10 K/min, and from 180 to 20 °C at a cooling rate of 10 K/min. Second cycle: from 20 to 280 °C at a heating rate of 10 K/min. For all DSC analysis, the result was given based on second cycle.

Wide angle X-ray diffraction (WAXD). Powder X-ray diffraction data were collected on a EMPYREAN diffractometer with Cu KR radiation (λ = 1.54056 Å) over the 2θ range of 5-80° with a scan speed of 0.128548/s and a step size of 0.0083556^o at room temperature. The samples were

crystallized isothermally at designed temperature for 2 h and measured at room temperature in air atmosphere.

Synthesis of catalyst. The di-nuclear cobalt catalyst *racemic*-**1** were prepared according to the literature method.[1]

Synthesis of cocatalyst. The cocatalyst PPN-DNP (PPN =bis(triphenylphosphine)iminium, DNP = 2, 4-dinitrophenoxide)[2] and PPN-PF_6 (PPN =bis(triphenylphosphine)iminium, PF_6 = hexafluorophosphate)[3] were prepared according to the literaturemethod.

Supplementary References.

1 Liu, Y., Ren, W. M., Liu, J. & Lu, X. B. Asymmetric copolymerization of CO_2 with *meso*-epoxides mediated by dinuclear cobalt(III) complexes: unprecedented enantioselectivity and activity. *Angew. Chem. Int. Ed*. **52**, 11594–11598 (2013).

2 Wu, G. P., Wei, S. H., Lu, X. B., Ren, W. M. & Darensbourg, D. J. Highly selective synthesis of CO_2 copolymer from styrene oxide. *Macromolecules* **43**, 9202–9204 (2010).

3 Liu, Y. *et al.* Mechanistic understanding of dinuclear cobalt(III) complex mediated highly enantioselective copolymerization of *meso*-epoxides with CO_2. *Macromolecules* **47**, 7775−7788 (2014).
